# Clinical Essays and Lectures

**Published:** 1903-03

**Authors:** 


**Clinical Essays and Lectures.** By HOWARD MARSH, F.R.C.S. Pp. xii., 303. London: J. & A. Churchill. 1902.

In this book the author has embodied various essays and lectures which from time to time have been published in the *St. Bartholomew's Hospital Reports* or elsewhere. They form a most agreeable collection of clinical and pathological material, which well repays a careful perusal. They are illustrated by numerous cases met with in the author's extensive experience, and give evidence of sound scientific knowledge, together with practical good sense.

